# Pyodermatitis–Pyostomatitis Vegetans: The Role of Langerin Deficiency in Disease Pathogenesis

**DOI:** 10.3390/jcm14124198

**Published:** 2025-06-12

**Authors:** Dan Pan, Jiongke Wang, Luyao Cai, Mei Huang, Qi Han, Zhijian Zheng, Xin Zeng, Qianming Chen, Ruixue Ai, Yu Zhou

**Affiliations:** 1 State Key Laboratory of Oral Diseases, National Clinical Research Center for Oral Diseases, Chinese Academy of Medical Sciences Research Unit of Oral Carcinogenesis and Management, West China Hospital of Stomatology, Sichuan University, Chengdu 610041, China; pandan@stu.scu.edu.cn (D.P.); wangjiongke@scu.edu.cn (J.W.); 2022324035035@stu.scu.edu.cn (L.C.); 2022224035071@stu.scu.edu.cn (M.H.); zengxin@scu.edu.cn (X.Z.); qmchen@scu.edu.cn (Q.C.); 2 State Key Laboratory of Oral Diseases, National Clinical Research Center for Oral Diseases, Department of Oral Pathology, West China Hospital of Stomatology, Sichuan University, Chengdu 610041, China; hanqi992011@scu.edu.cn (Q.H.); zhengzhijian@scu.edu.cn (Z.Z.); 3 Department of Clinical Molecular Biology, University of Oslo and Akershus University Hospital, 1478 Lørenskog, Norway

**Keywords:** langerin, Pyodermatitis–pyostomatitis vegetans, innate immune, adaptive immune, dendritic cells

## Abstract

**Background/Objectives:** Pyodermatitis–pyostomatitis vegetans (PPV) is a rare, chronic inflammatory mucocutaneous disorder. However, the etiology of PPV remains controversial. **Methods**: A review of online PPV case studies from PubMed, Wanfang database, Web of Science, and books has been performed. Comparative analysis of langerin expression has been conducted to verify the hypothesis summarized from the literature review by Immunohistochemistry (IHC). **Results**: A total of 63 patients were analyzed across 5 reviews, 44 case reports, and 1 book chapter. Our findings revealed distinct immunological alterations in PPV patients. Innate immunity was upregulated, marked by increased neutrophil and eosinophil counts and enhanced macrophage activity. Adaptive immunity was suppressed, with reduced dendritic cell (DC) numbers and activity and diminished adaptive immune responses. We hypothesize that langerin was a critical factor, contributing to adaptive immune suppression and a compensatory innate immune hyperactivation. **Conclusions**: We propose the hypothesis that langerin expression on Langerhans cells (LCs) plays a pivotal role in PPV pathogenesis by shifting the immune balance toward innate hyperactivation at the expense of adaptive immunity.

## 1. Introduction

Pyodermatitis–pyostomatitis vegetans (PPV), including Pyodermatitis vegetans (PDV) and pyostomatitis vegetans (PSV), is defined as a type of rare mucocutaneous disease [1]. PSV is characterized by multiple pustular erosions, exhibiting the typical “snail track” appearance on the oral mucosa, whereas PDV presents with vegetating plaques, typically involving the vulva, perianal region, scalp, neck, and axilla [2]. PPV significantly impacts patients’ quality of life, particularly when it originates in the oral cavity, the entry point of the digestive tract, impairing the ability to eat and absorb nutrients. However, the etiology of PPV remains controversial.

Inflammation reaction has been identified in PPV as the formation of abscesses by neutrophil and eosinophil [3] and the presence of Interleukin-6 (IL-6), Interleukin-8 (IL-8), and Tumor Necrosis Factor-alpha (TNF-α) [4]. Increased antibodies against bullous pemphigoid 230 and 180 (BP230 and BP180) are generally considered to contribute to bullous pemphigoid, and interestingly, BP230 and BP180 antibodies were found in PPV patients [5]. The detection of these antibodies, along with IgA deposits, suggests an autoimmune component in the pathogenesis of PPV, as proposed by several research studies [6]. The 70% frequent association of PPV with inflammatory bowel disease (IBD), the disease severity of PPV parallels that of IBD, and the observation that IBD treatment can alleviate PPV symptoms have led some researchers to consider PPV as a potential marker of IBD [7,8]. Hypersensitivity reactions between intestinal and oral mucosal antigens have been implicated in the pathogenesis of PPV [9]. Furthermore, some researchers have suggested that unidentified antigens may play a potential role in immunological dysfunction [10]. However, is there any mechanistic linkage underlying these inflammatory responses?

Immune dysfunction is a hallmark of PPV, characterized by enhanced innate immunity and impaired adaptive immunity, as summarized from the existing literature. We hypothesize that langerin is a pivotal molecule in PPV, mediating antigen presentation, innate–adaptive immunity crosstalk, and demonstrating lesion-specific expression. Its absence can explain PPV’s clinicopathological features, supported by wet-lab findings. Focusing on langerin could provide insights into PPV diagnosis and offer a novel therapeutic approach, such as Langerhans cell-targeted immunotherapy.

## 2. Materials and Methods

### 2.1. Literature Research

To investigate the altered molecules in PPV, retrospective research was implemented by research through online websites, including PubMed, Web of Science, and Wanfang Data. “Pyodermatitis pyostomatitis vegetans”, “Pyodermatitis vegetans”, and “pyostomatitis vegetans” were defined as terms, as a result of 43 years from 1981 to 2024. After reviewing titles, keywords, abstracts, and full text, 5 reviews, 44 cases, and 1 book chapter were included, with 63 patients in total, and the PRISMA structure is shown (Supplementary Figure S1).

### 2.2. Inclusion and Exclusion Criteria

Inclusion Criteria: ① Clinical Diagnosis: Cutaneous and/or mucosal lesions consistent with proliferative pyostomatitis/pyodermatitis; ② Histopathological Confirmation: Biopsy demonstrating neutrophilic and eosinophilic infiltration, epithelial hyperplasia, and/or microabscesses; ③ Associated Conditions: Patients with concurrent IBD may be included; ④ Direct and indirect immunofluorescence (DIF/IIF): Exclude bullous disorders; and ⑤ Age: No age restrictions

Exclusion Criteria: ① Alternative Diagnoses: Oral or cutaneous manifestations attributable to other conditions; ② Infectious etiologies; ③ Drug-induced stomatitis/dermatitis or trauma-related lesions; ④ Active Systemic Infections: Patients with uncontrolled systemic infections unless the infection is directly associated with the lesion pathology; ⑤ Immunosuppression: Use of high-dose immunosuppressive therapy (for conditions unrelated to PPV); and ⑥ Incomplete Data: Lack of histopathological confirmation or insufficient clinical documentation for accurate diagnosis.

### 2.3. Data Extraction and Quality Assessment

All articles were initially reviewed based on title and abstract, followed by a full-text screening to assess whether the cases met our inclusion criteria. Finally, an expert evaluated the quality of each case, considering whether the case could be definitively diagnosed as PPV and whether sufficient information was provided regarding patient characteristics and clinical setting.

### 2.4. Immunohistochemistry

The research gained approval by the Ethics Committee of the West China Hospital of Stomatology (No. WCHSIRBD-2024-333). After obtaining informed consent, we collected biopsy specimens from two patients with histologically confirmed PPV, three patients with clinically and histologically diagnosed oral lichen planus (randomly selected), and four healthy controls (normal oral mucosa, confirmed by histopathology). Formalin-fixed, paraffin-embedded tissue sections of the above tissues were obtained from our hospital’s pathology department for immunohistochemical staining. Tissue sections were deparaffinized by washing twice in distilled water. Antigen retrieval was performed by incubating the sections in sodium citrate buffer (pH 6.0) for 3 min under controlled heating. Subsequently, the sections were incubated overnight at 4 °C with a primary antibody against langerin (ab192027, Abcam; dilution 1:200, Cambridge, UK). After washing with PBS, a secondary goat anti-rabbit antibody (ZB-2301, ZSGB-Bio, Beijing, China) was applied for 1 h at room temperature. Immunoreactivity was visualized using a 3,3′-diaminobenzidine (DAB) chromogenic kit (ZLI-9017, ZSGB-Bio, Beijing, China) with a 1 min development time. Stained sections were examined under a light microscope, and images were captured for further analysis. Positive langerin expression was detected in the cytoplasm.

### 2.5. Data Analysis

Statistical analyses were achieved by SPSS 27.01 software and Graphpad 9.0 through the chi-square test as well as Student’s unpaired tests.

## 3. Summary from Literature Research

### 3.1. Current Status of Clinical Features, Pathology, and Treatment of PPV

**Figure 1:**
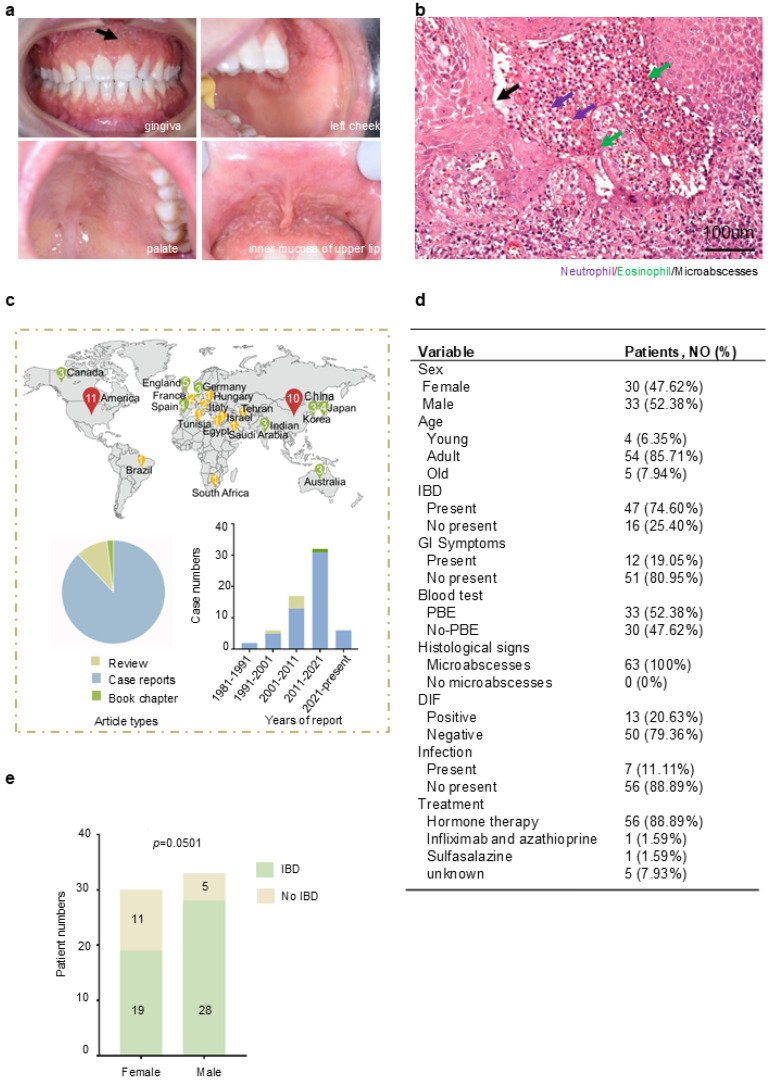
Reveal typical clinical and histological features of PPV: (**a**): clinical manifestation, extensive yellowish-white erosions, formation of pseudoepitheliomatous hyperplasia, and “snail track” lesions (black arrow) on the gingiva, left cheek, palate, and inner mucosa of upper lip. (**b**): Histological manifestation (H&E stain, original magnification ×200 μm); Infiltration of neutrophil (purple arrows) and eosinophil (green arrows) formed microabscesses (black arrows). One patient with PSV in the West China School of Stomatology, Sichuan University. (**c**): The distribution of PPV in different countries was shown in map, and the number inside the icon indicates the number of cases reported in that country. Article types of PPV cases and years of case reports were summarized. (**d**): Summary of 63 cases’ clinic features in PPV. (**e**): Analysis of sex difference and concomitant with IBD in PPV (*p* = 0.0501). Additional data are in (Supplementary Figure S1). Data are presented as mean ± S.E.M. Group differences were analyzed with the chi-square test.

Oral mucosa is the most frequently involved site of PPV [11]. The typical clinic presentations of PPV [12] include mucosal congestion, sterile pustules, pseudoepitheliomatous hyperplasia, and the formation of distinctive “snail track” lesions (Figure 1a), as well as erythematous papules on the skin. Histopathologically, PPV is marked by an inflammatory infiltrate predominantly consisting of neutrophils and eosinophils, with microabscesses observed in both the epidermis and dermis (Figure 1b). The diagnosis of PPV relies on typical clinical manifestations, increased eosinophil counts in the blood, and, crucially, histopathological evidence. The diagnosis of PPV also requires the exclusion of bullous disorders through DIF/IIF.

Oral prednisolone achieves excellent therapeutic outcomes in most PPV patients, but it is not suggested due to the side effects of prednisolone for long-term use. Thus, sulfasalazine, the combination of azathioprine and infliximab, has also been proposed as an effective and safe treatment for mild and moderate PPV patients [13], while only several patients received below treatment and the effectiveness remains to be further observed. Moreover, the use of sulfasalazine presents hepatotoxicity, while azathioprine shows obvious allergic reactions, tremor, and teratogenicity [14,15]. The clinical use of infliximab is associated with several well-documented adverse effects, including immunosuppression, infusion-related reactions, and abdominal pain [16]. Thus, it is necessary to explore PPV’s mechanism to propose target therapies.

To investigate the altered molecules in PPV, retrospective research was implemented by research (Supplementary Figure S1). The distribution of PPV in different countries was shown in a map (Figure 1d, top). These cases mainly occurred in the Americas, Asia, and Europe. It might be due to the current limited understanding of this disease, resulting in misdiagnosis and missed diagnosis in other countries. The publications were summarized according to the publication types and year information (Figure 1c, bottom). This refers to the understanding that PPV is still limited to case reports through clinical manifestations, but barely any mechanism, with only a few review summaries. As for the publication time, the bar chart showed that the incidence of PPV was gradually increasing, especially in the last 10 years. However, there has been a lack of comprehensive induction and summary of PPV in the past decade. A comprehensive review to summarize the potential mechanism of PPV is needed.

The clinic characteristics of these patients are detailed in (Figure 1d), which includes sex, age, presence of IBD, gastrointestinal symptoms, peripheral blood eosinophilia (PBE), histological signs, results from DIF/IIF, concomitance with infection, and treatment. We attempted to clarify the underlying relationship of each clinic’s features according to the data we collected. Our research shows the female and male composition ratios of PPV are 47.62% and 52.38%, respectively. The majority of affected individuals were adults (85.71%), with a mean age at diagnosis of 38.05 years. Notably, 74.60% of patients with PPV are concomitant with IBD, which is in line with the previous study [1,3], and 19.05% of patients present gastrointestinal symptoms. A total of 52.38% of PPV patients present PBE in blood. All PPV patients show the formation of microabscesses. A total of 79.36% of patients present DIF negative. A total of 11.11% of patients of PPV concomitant with bacterial infection, predominantly with *Staphylococcus aureus*. Hormone therapy has shown a significant therapeutic effect on 56 patients, while a case with infliximab treatment also shows a beneficial choice [17], and oral sulfasalazine may serve as an effective adjunctive therapy [13]. We then analyzed the correlation of these clinical parameters. There is a sign indicating male patients with PPV tend to be concomitant with IBD more than female patients (Figure 1e, *p* = 0.0501). The other clinic parameters have no significant differences (Supplementary Figure S2).

### 3.2. Enhancement of Innate Immunity and Weakening of Adaptive Immunity in PPV

**Figure 2:**
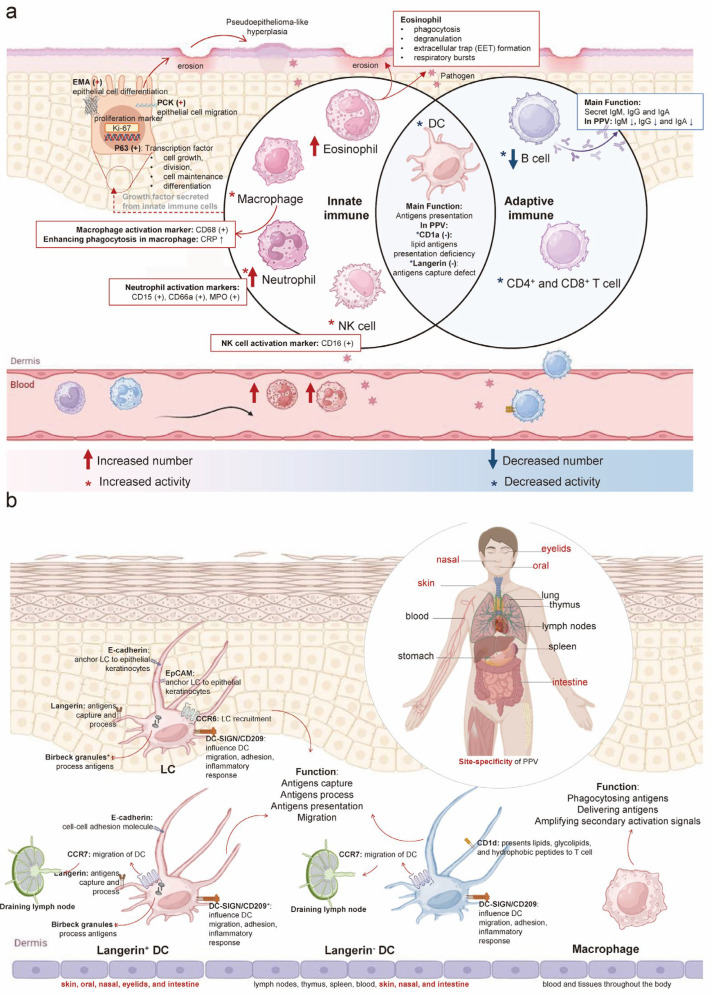
Langerin deficiency results in enhancement of innate immunity and weakening of adaptive immunity in PPV: (**a**): In PPV, innate immune cells such as eosinophils, macrophages, neutrophils, NK cells, and DC show increased numbers and/or activity, except for DC. Adaptive immune cells, including DC, B cells, and T cells, exhibit decreased numbers and activity. CD68, a marker of macrophages, is positive along with increased CRP, enhancing macrophage phagocytosis and indicating increased macrophage activity. CD15, CD66a, and MPO are positive as neutrophil markers, reflecting neutrophil activation. CD16 positivity indicates NK cell activation. CD1a and langerin are negative, both considered DC markers. A reduction in IgA, IgM, and IgG antibodies produced by B cells in serum suggests decreased B cell activity and numbers in PPV. Positive PCK (regulating cell migration), EMA (regulating epithelial cell differentiation), P63 (regulating cell proliferation), and Ki-67 (cell proliferation marker) are observed in response to recurrent erosion and epithelial hyperplasia lesions of PPV on the skin and mucosa. (**b**): Four types of APCs are found in humans: LC, langerin^+^ DC, langerin^−^ DC, and macrophage. LC and langerin^+^ DCs are distributed in the skin, oral cavity, nasal passages, eyelids, and intestines. Langerin^−^ DCs are found in lymph nodes, thymus, spleen, blood, skin, nasal passages, and intestines. Macrophages are distributed throughout the blood and tissues of the body. LC, langerin^+^ DC, and langerin^−^ DC share functions like antigen capture, processing, presentation, and migration. Besides phagocytosing particulate antigens, macrophages enhance the immune response by delivering antigens and amplifying secondary activation signals. The expression of langerin, CD1a, Birbeck granules, E-Cadherin, Epithelial-cell adhesion molecule (EpCAM), CC-chemokine receptor 6 (CCR6), CC-chemokine receptor 7 (CCR7), CD1d, and DC-SIGN shown in four types of APCs. (CRP: C-reactive protein; MPO: Myeloperoxidase; PCK: Pan cytokeratin; EMA: Epithelial membrane antigens; APCs: Antigen-presenting cells).

Our study aimed to identify immunological factors and underlying mechanisms. We summarized key markers with emphasis on their expression and function (Figure 2a). Innate immunity analysis showed CD68 (PG-M1) positivity, primarily expressed in monocytes and macrophages [18], demonstrating macrophage activation. CRP, an acute-phase protein, significantly increases during infection or tissue damage [19] and enhances complement activation and macrophage phagocytosis to clear pathogens and damaged cells [20].

Histopathological analysis revealed elevated eosinophils and neutrophils. Eosinophils (12.9%; normal 0.5–5%; absolute count 1.2 × 10^9^/L, normal 0.02–0.5 × 10^9^/L) [21] function as cytotoxic effector cells via phagocytosis, degranulation, and extracellular trap formation [22,23]. Neutrophils, the primary blood phagocytes, rapidly migrate to inflammatory sites and deploy these traps to ensnare and eliminate pathogens through cell death mechanisms [24]. Except for the increased neutrophil number observed, neutrophil markers, CD15, CD66a, and myeloperoxidase (MPO) positive were detected, whose expression levels were indicative of neutrophil functionality [25,26]. As a typical marker of neutrophils, myeloperoxidase possesses a potent antimicrobial property that is effective against ingested bacteria [27]. CD16 was detected in PPV. CD16 is recognized as a surface marker on NK cells, linked to the activation of these cells [28]. These innate immune cell alterations represent a critical component of the body’s defense against pathogens while minimizing tissue damage.

In adaptive immunity, reduced IgA, IgM, and IgG levels indicate impaired B-cell function. Additionally, langerin and CD1a were absent. Langerin, a type II lectin receptor expressed on DC, plays a role in antigen capture, internalization, and processing for presentation [29]. CD1a, a member of the CD1 family of transmembrane proteins and a marker for LC, is involved in presenting lipid antigens to T cells [30].

Epithelial cell markers PCK, EMA, P63, and Ki-67 (MIB-1) were positive. Notably, 20% of proliferating squamous epithelium in PPV patients showed Ki-67 (MIB-1) positivity. PCK, the primary cytoskeletal protein in keratinocytes, maintains epithelial integrity and regulates cellular growth and migration [31]. EMA, a type I membrane protein co-expressed with PCK [32], serves as an epithelial marker and regulates epithelial differentiation. P63 positivity indicates its role as a transcription factor governing epithelial regeneration [33], with regulatory functions in cell growth, differentiation, adhesion, and apoptosis through DNA binding [34,35]. Ki-67 protein, present in all active phases of the cell cycle, is seen as a proliferation marker [36], and the Ki-67 positive index is used to determine epithelial cell proliferation capacity [37]. The positivity for PCK, EMA, P63, and Ki-67 likely reflects recurrent epithelial erosion and hyperplasia in PPV lesions, a process involving epithelial proliferation and migration.

## 4. Hypothesis from Literature Research

### 4.1. PPV Is an Inflammatory Disease Mediated by Langerin Deficiency

Given the observed inhibition of adaptive immunity, DC and the activation of the innate immune response, we hypothesize that the disorder may originate from disruptions in the initiation phase of adaptive immunity.

DCs, recognized as the most proficient antigen-presenting cells (APCs) [38], and macrophages, another type of APC, are pivotal in detecting and presenting antigens, in resolving skin inflammation [39] (Figure 2b). The skin hosts two major populations of langerin^+^ DC: LC in the epidermis and langerin^+^ DC in the dermis [40]. Langerin^+^ DC is consistent with the site-specificity observed in PPV. The skin also contains two additional subpopulations of APCs: langerin^−^DC and macrophage, which share functional similarities with langerin^+^ DC [41]. However, the systemic distribution of langerin^−^DC and macrophages, without apparent tissue specificity, suggests the possibility that both epidermal LC and dermal langerin^+^ DC may play essential roles in PPV pathogenesis.

The cellular markers distinguishing APCs (langerin^−^ DC and langerin^+^ DC) were summarized, including langerin, CD1a, Birbeck granules, E-Cadherin, epithelial cell adhesion molecule (EpCAM), CC-chemokine receptor 6 (CCR6), CC-chemokine receptor 7 (CCR7), CD1d, and DC-SIGN [40] (Figure 2b; Table 1). Determining which of these markers plays a pivotal role in disease mediation remains a critical research question. Notably, langerin^−^a type II C-type lectin receptor expressed by LC and langerin^+^ DC in the skin and mucosal tissues [42,43], correlates with the primary sites of PPV. Langerin has been viewed as a receptor involved in both innate and adaptive immunity. CD1a, as a member of the MHC class I antigen family, presents lipid antigens to T cells to mediate adaptive immunity [44]. In the process of LC presenting antigens to CD1a-restricted T cells, the dispensable role of langerin in capturing antigens has been verified [45], so we supposed that the process of lipid antigen presentation of CD1a is probably downstream of langerin-mediated antigen capture. Moreover, langerin can capture antigens and subsequently internalize them into Birbeck granules to degrade [40], and is seen as an inducer of the formation of Birbeck granules. Birbeck granules, exclusive to LC, are implicated in receptor-mediated endocytosis and antigen presentation [46]. The presence of Birbeck granules appears to be a direct result of langerin’s antigen-capturing function.

Human epidermal LC consistently express E-cadherin, a homotypic adhesion molecule that anchors LC to neighboring keratinocytes [47]. EpCAM is an isotypic adhesion molecule found on human LC [48]. The primary roles of E-cadherin and EpCAM are to anchor LC to epithelial keratinocytes, influencing LC distribution. While E-cadherin expression is downregulated, potentially leading to LC detachment from keratinocytes, EpCAM levels remain stable [49]. The chemokine receptors CCR6 and CCR7 are implicated in LC migration, with CCR6 directing pathologic LC to non-lymphoid tissues like skin and bone [50], and CCR7 being crucial for LC transit to the skin-draining lymph node [51]. Although CCR6 and CCR7 are expressed in skin and mucous membranes, consistent with PPV sites, they primarily influence LC localization rather than directly modulating adaptive or innate immunity. CD1d, a member of the cell surface glycoprotein CD1 family, presents lipids, glycolipids, and hydrophobic peptides to T cells [52]. Its expression in the skin is largely confined to dermal DC and epidermal keratinocytes. While CD1d participates in antigen presentation, it is not site-specific, and no expression anomaly was found, so it may not be the primary functional protein in this context. DC-SIGN, a C-type lectin receptor, is expressed on human dermal DC and myeloid APC [53]. It plays a significant role in DC migration, adhesion, inflammatory response, T cell activation, immune response initiation, and evasion of immune detection by pathogens and tumors [54]. DC-SIGN’s distribution on dermal dendritic cells allows for its presence across various body sites.

Since langerin is essential for antigen capture and its expression pattern correlates with PPV lesion distribution, the observed langerin deficiency in PPV may explain the compensatory innate immune enhancement and impaired adaptive immunity. This suggests langerin^+^ LC plays a pivotal role in PPV pathogenesis. Below, we will discuss the homeostasis and function of human LC and other langerin^+^ DC to further elaborate on this hypothesis.

### 4.2. Enhanced Innate and Inactive Adaptive Immune Response Due to Langerin Deficiency in PPV

**Figure 3:**
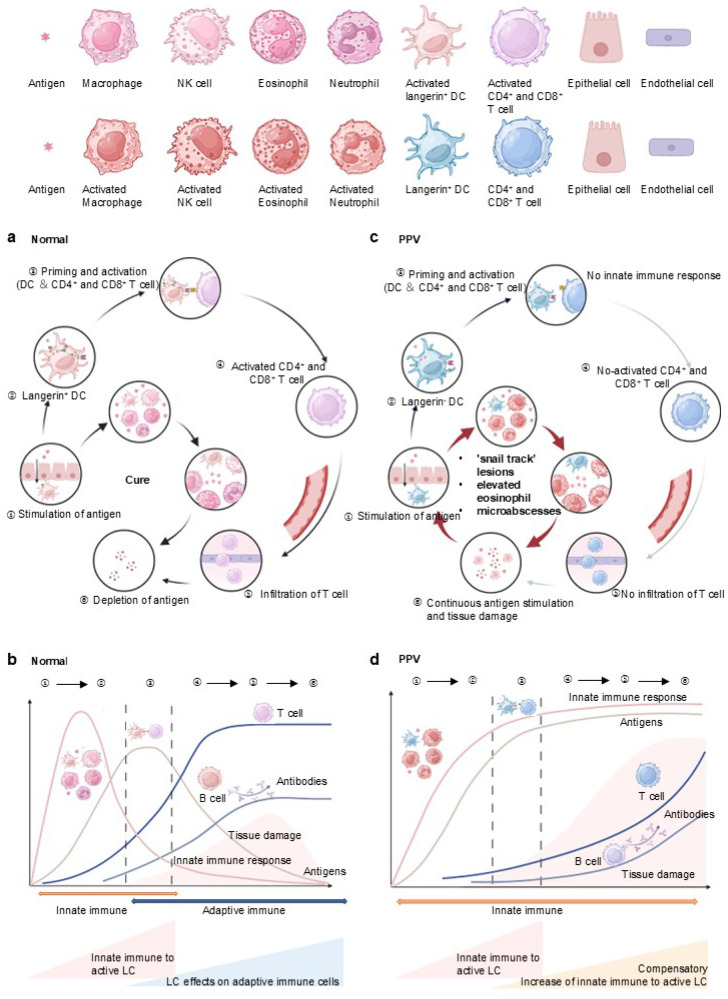
Immune response to antigens in NEP and enhancement of innate immunity in response to langerin deficiency in PPV: (**a**) Under normal circumstances, innate immune cells, including macrophages, NK cells, eosinophils, and neutrophils, serve as the first line of defense against pathogen invasion, prompting the adaptive immune process. Antigens are internalized into Birbeck granules in DC for processing and presented to T cells. Following this, CD4^+^ and CD8^+^ T cell is activated, marking the onset of adaptive immunity and T cell infiltration. The antibodies secreted by B cells, along with the effector and cytotoxic responses released by T cells, eliminate antigens and maintain a normal physiological state. (**b**): As the adaptive immune response strengthens, the role of the innate immune system gradually diminishes, and pathogens are progressively eliminated. The pathogen load peaks during antigen presentation and gradually decreases until it disappears with the activation of adaptive immunity. In the early stages of the immune response, tissue damage gradually increases, but with the advent of adaptive immunity, tissue damage is repaired. (**c**): In the case of PPV, due to the lack of langerin, DCs fail to present antigens to CD4^+^ and CD8^+^ T cells. This failure interrupts T cell activation and infiltration, leading to decreased antibody secretion by B cells and reduced effector and cytotoxic responses by T cells. (**d**): Concurrently, the weakened adaptive immunity prompts a compensatory enhancement of the innate immune response, evidenced by increased macrophage, eosinophil, and neutrophil. Due to the weakening of adaptive immunity in PPV, antigens present a stronger response from the innate immune system. The persistent antigens cannot be eliminated due to the dysfunction of the adaptive immune system, leading to accumulated tissue damage.

Under normal circumstances (Figure 3a,b), innate immune cells are the first line of defense against pathogen invasion and prompt the adaptive immune process [55]. Macrophage primarily performs phagocytic and digestive functions against antigens, while NK cells release lytic particles containing molecules like perforin, granzyme, and granulin, which induce the death of target T cells. Eosinophil exerts the effect of killing antigens and releasing the contents of the granules, which can cause tissue damage and promote the progression of inflammation. Neutrophil is the main phagocytic cell in the blood and first reaches the site of inflammation when infection occurs, after which neutrophil extracellular traps (NETs) are formed to trap and kill pathogens concomitant with cell death. DCs exert the effect of elimination, phagocytosis, and dissolution to process antigens in the innate immune system, while DCs are dispensable in antigen presentation to T cells and in activating the adaptive immune system [56]. In the adaptive immune response, antigens are internalized into Birbeck granules for processing and subsequent presentation to T cells by DC. After that, T cell is activated with the onset of adaptive immunity, and infiltration of T cells in the skin and mucosa is formed. Then the role of the innate immune system gradually weakens, and pathogens are gradually eliminated. The process of priming and activation of T cells also releases cytokines to enhance the innate immune system, such as IFN-γ, IL-2 to stimulate macrophages, and IL-5 to activate eosinophils. B cell-derived antibodies and T cell-mediated effector/cytotoxic responses clear antigens, restoring physiological homeostasis (Figure 3a). The pathogen peaks in infection at the time of antigen presentation and gradually decreases until it disappears with adaptive immune activation. In the early stages of the immune response, tissue damage gradually increases, and with the advent of adaptive immunity, tissue damage repairs itself (Figure 3b).

However, in PPV, the pathogen enters and acts on innate immune cells, while DCs fail to present antigens to T cells, given the lack of langerin (Figure 3c,d). It induces the interrupt to activate T cells and infiltration of T cells, followed by a decrease in antibodies secreted by B cells and effector and cytotoxicity released by T cells. Concurrently, the weakened adaptive immunity prompts a compensatory enhancement of the innate immune response, evidenced by increased macrophage, eosinophil, and neutrophil, aligning with the histopathological findings in PPV (Figure 3c). Due to the weakening of adaptive immunity in PPV, antigens can be more responded to by innate immunity, causing a compensatory enhancement of innate immunity. The persistence of the antigens cannot be eliminated, causing accumulated tissue damage (Figure 3d).

### 4.3. Immunohistochemical Staining Revealed Langerin Negative in PPV

To validate our hypothesis, we conducted langerin immunohistochemical staining on epithelial samples, including four normal (NEP), three oral lichen planus (OLP), and two PPV cases. As an immune-mediated inflammatory disorder, OLP demonstrates significantly elevated langerin expression compared to healthy tissue [57,58], reflecting immune system activation. Thus, OLP individuals were used as a positive control.

**Figure 4:**
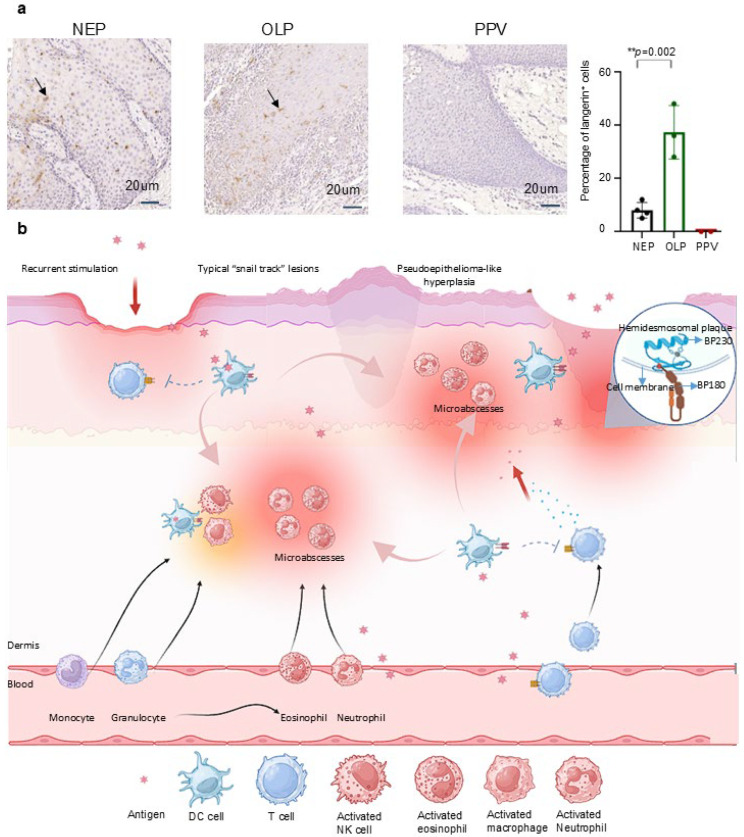
Identification of langerin negative in PPV interprets the clinicopathological manifestations of PPV: (**a**): Four NEP, three OLP, and two PPV tissues were used to stain langerin. The langerin expression in PPV was barely compared to NEP, with an 8% decrease. Langerin expression was increased by about 29% in patients with OLP compared with NEP (** *p* = 0.002, 29.330 ± 1.156). The expression of langerin in PPV was reduced by about 30% compared with that in OLP. Scale bar = 20 μm. Data are presented as mean ± S.E.M.; ns: no statistical significance; ** *p* ≤ 0.01. Group differences were analyzed with Student’s unpaired tests. (PPV: Pyodermatitis–pyostomatitis vegetans; NEP: Normal epithelium; OLP: Oral lichen planus). (**b**): Antigens cross epithelial and endothelial cells to stimulate the innate immune system. The lack of langerin impairs adaptive immune function, leading to a compensatory increase in innate immunity. Neutrophil migrates from the bloodstream to lesion sites, where the infiltration of eosinophils and neutrophils forms microabscesses. Recurrent stimulation by inflammatory factors and antigens leads to the formation of damaged epithelial cells, clinically presenting as nodular hyperplasia and pseudoepitheliomatous hyperplasia. Additionally, the recurrent cycle of lesion stimulation and healing, along with the fusion of damaged and adjacent normal epithelial cells, results in the characteristic “snail track” lesions in PPV. Due to epithelial inflammation, damage to the basal layer, BP180 and BP230 antigens in basement membrane area are exposed to dermis, and BP180 and BP230 antibodies are produced as a result to produce.

As we can see, the expression of langerin in OLP (37.33%) is higher than that in NEP (8.00%) and negative in PPV (0.00%) (Figure 4a). Langerin expression increased by approximately 29.33% in OLP patients compared to NEP (** *p* = 0.002). Conversely, langerin expression in PPV was nearly absent, showing an 8% decrease compared to NEP and a 37.33% reduction compared to OLP. It has been reported that expression of langerin was increased under inflammatory states [59]. Our data show that langerin failed to increase, or possibly decrease, the response to antigen stimulation in inflammatory states in PPV. This result showed a lack of langerin in PPV patients. Combining the above arguments, we verified that langerin is a key pathogenic factor in PPV. However, although the results appear to support our hypothesis, the statistical power is limited due to the small sample size, which is attributed to the rarity of PPV.

### 4.4. The Deficiency of Langerin Interprets the PPV Clinical Pathological Manifestations

Besides, langerin accounts for the overall clinical and pathologic manifestations of PPV (Figure 4b) from the aspect of immune dysfunction. Antigens, whether foreign or from the bloodstream, cross epithelial and endothelial cells to stimulate DC. The lack of langerin impairs the adaptive immune function, leading to a compensatory increase in innate immunity. This is reflected in the elevated eosinophil counts found in the blood, epithelial, and mucosal tissues of PPV patients. Neutrophils migrate from the bloodstream to the lesion sites, where the infiltration of eosinophils and neutrophils forms microabscesses. The recurrent stimulation by inflammatory factors and antigens leads to the formation of damaged epithelial cells. Moreover, the recurrent cycle of lesion stimulation and healing presents clinically as nodular hyperplasia and pseudoepitheliomatosis. Alongside the fusion of damaged and adjacent normal epithelial cells, this results in the characteristic “snail track” lesions seen in PPV. This hypothesis supports the notion that langerin deficiency underlies the etiology of PPV, explaining its clinical and pathological manifestations. Based on our hypothesis, we proposed a new sight to the mechanism, diagnosis, and treatment (Table 2).

## 5. Discussion

In this work, based on the observed changes in clinical indicators of PPV, we reviewed all current PPV patients and summarized their clinical and pathological characteristics, and discovered the unique molecule langerin. We hypothesized that langerin deficiency is a primary contributor to PPV, due to impairments in the langerin-mediated antigen presentation process, which leads to the weakening of adaptive immunity and the compensatory enhancement of innate immunity, revealing a new possible mechanism for the etiology of PPV. Furthermore, to test our hypothesis, we analyzed tissue samples from PPV patients for langerin expression, and the samples exhibited a marked langerin deficiency. In addition, the etiological mechanism of langerin deficiency can explain the clinical and pathological manifestations of current PPV patients, which further validates our hypothesis.

Ulcerative colitis, chronic relapsing inflammatory diseases, and Crohn’s disease are included in IBD. Dysfunction of the immune response contributes to IBD, and DC is crucial in the etiology of IBD [5]. The pathogenesis of IBD is intricate, including genetics, individual predisposition, immunity, et al. There is a lack of research on the pathogenesis link between PPV and IBD. As some researchers consider PPV a reminder of active IBD [60], 74.60% of patients with PPV are concomitant with IBD in our study (Figure 1e). After the treatment of IBD, the alleviation of PPV symptoms was discovered in PPV patients concomitant with IBD, which further suggests the correlation of PPV and IBD. Though certain researchers hold that PPV is an extra-intestinal manifestation of IBD, some PPV patients without IBD or related symptoms, and PPV even occurs in patients after total colectomy [60]. PPV is frequently associated with IBD, but classifying PPV as the extraintestinal manifestation of IBD is also not entirely convincing. The result that male patients with PPV tend to have concomitant IBD more than female patients (Figure 1e) may be explained by the small sample size of samples or male PPV patients are more deficient in langerin, considering the key pathogenic factor of langerin in PPV. In conclusion, there is a possibility that langerin is also involved in the pathology of IBD in its special way. However, this hypothesis requires further experimental and clinical validation.

The positivity of BP230 and BP180 antibodies might be due to epithelial inflammatory damage to the basement membrane in certain patients in response to BP230 and/or BP180 antigens in the dermis. Though inflammatory damage essentially invades the dermis in all PPV patients, only a few patients presented positive for BP230 and BP180 antibodies, which might account for these patients possessing an autoimmune response rather than others. However, more PPV patients and experiments need to be confirmed.

The limitations of this project are determined by the small number of clinical samples, because PPV is a rare disease with only 63 patients included from the studies published over the last 34 years. Due to challenges in patient recruitment, only two samples were ultimately available for analysis, both of which confirmed negative langerin expression. In addition, further cellular and animal experiments are needed to test our hypothesis. Larger samples are needed to validate the hypothesis and use animal experiments with cells and langerin knock-out mice (langerin DTR mice) [61] to detect the role of langerin in PPV. However, the hypothesis can explain the reported 63 cases and provide new insights into this disease. Alternative mechanisms, such as epithelial damage triggering an immune response that subsequently downregulates langerin expression, should also be considered.

## 6. Conclusions

In conclusion, we propose the notion that PPV is mediated by the lack of langerin. We proposed a new hypothesis of the mechanism, diagnosis criteria, and treatment of PPV (Table 2) based current features of PPV. As a result of our discovery, we suggest that langerin may serve as a diagnostic marker for PPV. On the other hand, recent studies targeting skin LC with anti-langerin monoclonal antibodies for HIV-1 gp140 showed that it was possible to induce simultaneous T-cell- and B-cell-mediated immune responses [62]. Thus, the same approach could be considered for PPV, stimulating the shift of the immune response to adaptive immunity. However, the project needs numerous experiments to substantiate in vivo and in vitro. The related and solid experiments are still needed to illustrate the etiology of PPV and discover target drugs.

## Figures and Tables

**Table 1:** The distribution of langerin and Birbeck granules overlaps with PPV sites.

	LC	Langerin^+^ DC	Langerin^−^ DC	Macrophage	Location
CD1a	+	+	+	−	Epithelial cell, B cell, dendritic cells, and thymocyte
Langerin	+	+	−	−	**Skin, oral, nasal, eyelids, intestine, liver, lungs,** lymph nodes, thymus, and spleen
Birbeck granules	+	+	−	−	**Skin, oral, nasal, eyelids, and intestine**
E-cadherin	+	+	−	−	Epithelial cell
EpCAM	+	−	−	−	Epithelial cell
CCR6	+	−	−	−	All lymphatic and non-lymphoid tissues
CCR7	−	+	+	−	Blood, bone marrow, lymph nodes, and intestines
CD1d	−	−	+	−	All antigen-presenting cells and thymic cortex cells
DC-SIGN/CD209	+	+	+	−	Surface of immature dendritic cells
Location **Site-specificity** of PPV	**Skin, oral, nasal, eyelids, and intestine**	**Skin, oral, nasal, eyelids, and intestine**	Lymph nodes, thymus, spleen, blood, **skin, nasal, and intestinal**	Blood and tissues throughout the body	

The sites with bold emphasis are the same site of PPV: LC: Langerhans cell; DC: Dendritic cell.

**Table 2:** Langerin deficiency interprets PPV current clinical and pathologic discovery.

PPV	Current Status	New Insights
**Clinic** **manifestations**	Typical “snail track” lesions on the oral mucosa Pseudoepitheliomatous hyperplasia Erythematous papules on the skin	
**Mechanism**	Unknown etiology, hypothesis shown as below: (1) Inflammation reaction: microabscesses, IL-6, IL-8, TNF-α (2) Autoimmune processes: BP230, BP180 antibodies and IgA deposits (3) Hypersensitivity reaction with IBD: concomitance of PPV and IBD, the severity of PPV parallels that of IBD (4) Unknown antigens: immunological dysfunction, such as elevated neutrophil and eosinophil.	**Langerin deficiency mediated disease** (1) Langerin deficiency induces adaptive immunity inhibition and innate immune enhancement, as a result microabscesses, IL-6, IL-8, TNF-α were found. (2) Epithelial damage, as a result BP230/180 and IgA move to dermis to increase auto-immune response. (3) Male patients with PPV tend to concomitant with IBD more than female patients (Figure 1e), may be explained with the small size of samples or male PPV patients are more deficient in langerin inducing dysfunction of bowel. (4) Unknown antigens: immunological dysfunction, such as elevated neutrophil and eosinophil.
**Laboratory findings**	Elevated eosinophil counts in routine blood tests DIF/IIF: negative	
**Histopathological manifestations**	Infiltration of neutrophils and eosinophils	Infiltration of neutrophils and eosinophils. **Immunohistochemical staining: negative langerin**
**Treatment**	Hormone therapy Prednisolone sulfasalazine The combination of azathioprine and infliximab	**Langerin target** therapy to avoid the side effects of previous treatment

The bold part with emphasis on the new sights for our project in the broad view of PPV.

## Data Availability

The original contributions presented in the study are included in the article, further inquiries can be directed to the corresponding author.

## Supplementary Materials

The following supporting information can be downloaded at: https://www.mdpi.com/article/10.3390/jcm14124198/s1.
