# Supplementary material for: Pyodermatitis–Pyostomatitis Vegetans: The Role of Langerin Deficiency in Disease Pathogenesis
Source: J Clin Med. 2025 Jun 12;14(12):4198. doi: 10.3390/jcm14124198 (PMC12194030; doi:10.3390/jcm14124198)
Supplement: Supplementary file 1 [file jcm-14-04198-s001.zip › jcm-3598178-supplementary.pdf]

Supplementary Figures

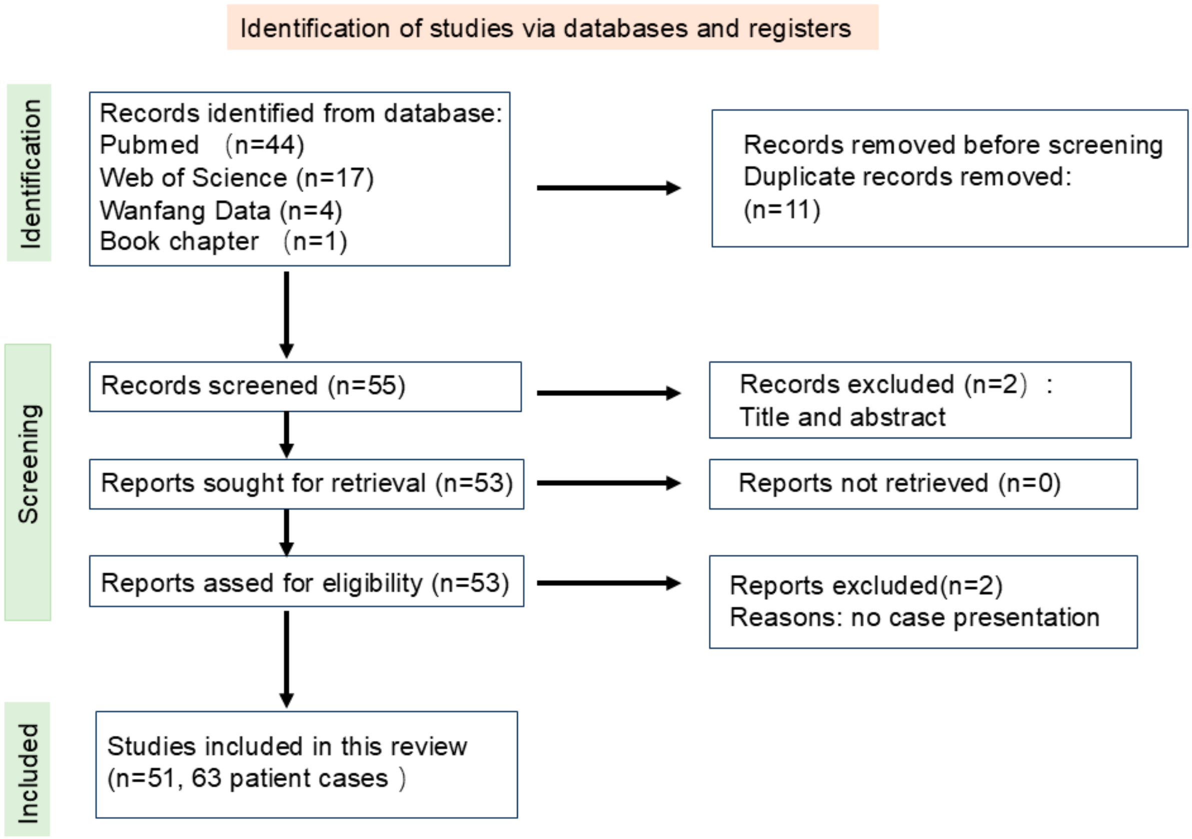

Supplementary Figure S1: PRISMA Flow Diagram. PRISMA: The terms 'pyodermatitis-pyostomatitis vegetans,' 'pyodermatitis vegetans,' and 'pyostomatitis vegetans' were identified through a comprehensive search spanning 1981 to 2024. Following a systematic review of titles, keywords, abstracts, and full texts, we included 5 review articles, 44 case reports, and 1 book chapter, encompassing a total of 63 patients.

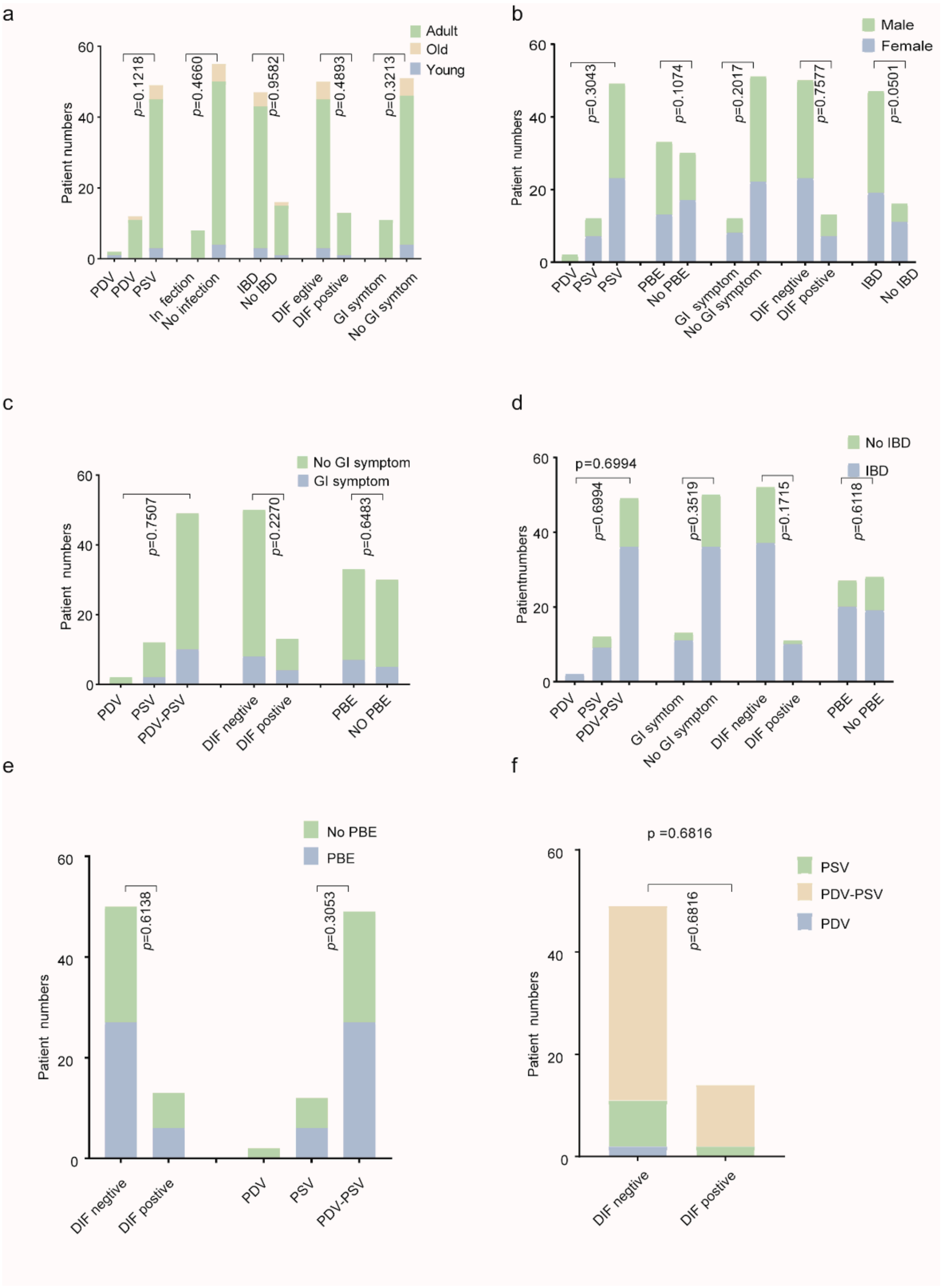

Supplementary Figure S2. (a–f): Correlation analysis between different clinical parameters. Data are presented as mean ± S.E.M. Group differences were analyzed with the chi-square test.
